# Sustained oxygen evolution reaction by 2D mesoporous polyoxometalate-derived composite electrocatalysts

**DOI:** 10.1039/d6sc02532c

**Published:** 2026-07-16

**Authors:** Rongji Liu, Yupeng Zhao, Archismita Misra, Dandan Gao, Adam H. Clark, Montaha Anjass, Johannes Biskupek, Ute Kaiser, Guangjin Zhang, Carsten Streb

**Affiliations:** a Johannes Gutenberg University Mainz, Department of Chemistry Duesbergweg 10–14 55128 Mainz Germany rongji.liu@uni-mainz.de dandan.gao@uni-mainz.de carsten.streb@uni-mainz.de; b Institute of Inorganic Chemistry I, Ulm University Albert-Einstein-Allee 11 89081 Ulm Germany; c Helmholtz-Institute Ulm, Electrochemical Energy Conversion Helmholtzstr. 11 89081 Ulm Germany; d Paul Scherrer Institut Forschungsstrasse 111 Villigen CH-5232 Switzerland; e University of Sharjah, Department of Chemistry PO Box 27272 Sharjah United Arab Emirates; f Central Facility of Electron Microscopy for Materials Science, Ulm University Albert-Einstein-Allee 11 89081 Ulm Germany; g CAS Key Laboratory of Green Process and Engineering, Institute of Process Engineering, Chinese Academy of Sciences 100190 Beijing China; h Bundesanstalt für Materialforschung und -prüfung (BAM), Department of Materials and the Environment Unter den Eichen 87 12205 Berlin Germany

## Abstract

The electrocatalytic oxygen evolution reaction (OER) is the bottleneck for sustainable water electrolysis to access green hydrogen as a carbon-neutral energy carrier. Here, we report the modular design of a noble metal-free composite OER electrocatalyst, which features high electrical conductivity, high OER reactivity and high durability. To this end, we present a new synthetic strategy where the Keggin-type polyoxomolybdate Ni[HPMo^VI^_12_O_40_] is used as the sole molecular precursor in a scalable top-down fabrication approach. This provides access to a high-performance OER composite electrocatalyst (*η*_10_ = 320 mV) where Ni metal clusters are deposited on η-MoC/MoO_2_ nanocomposites anchored on electrically conductive N, P-doped mesoporous carbon. The composite catalyst shows sustained OER activity in 1 M aqueous KOH solutions over prolonged periods (*t* > 20 h) at a low overpotential (*η* = 360 mV) and high faradaic efficiency (>95%). This new synthetic concept will enable the development of multifunctional (mixed) metal carbide/oxide composites as high-performance electrocatalysts for challenging energy conversion and storage reactions.

## Abstract 2

Polyoxometalate-derived composite electrocatalysts are reported which show sustained electrochemical oxygen evolution reaction performance (*η*_10_ = 320 mV, FE > 95%) comparable with noble metal-based reference compounds.

## Graphical abstract

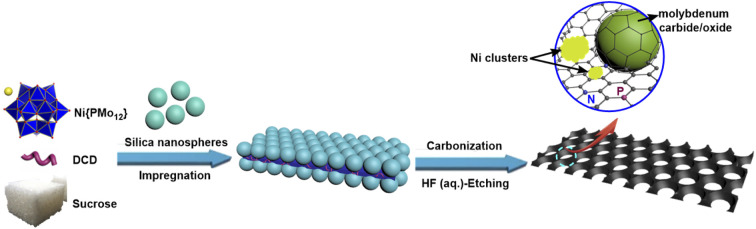

## Introduction

Water electrolysis into oxygen and hydrogen is a central pillar of sustainable carbon-neutral energy conversion technology. For efficient operation, water electrolysis requires the catalytic hydrogen evolution reaction (HER) at the cathode and the catalytic oxygen evolution reaction (OER) at the anode. Currently, the development of viable OER catalysts is a major bottleneck, as the OER is a challenging proton-coupled four-electron process, which often requires high overpotentials (compared to the thermodynamic electrochemical potential of 1.23 V *vs.* the reversible hydrogen electrode (RHE)).^1^ The use of noble metal-based OER catalysts such as IrO_2_ or RuO_2_ is a major limitation for large-scale industrial water electrolysis. In this context, earth-abundant transition-metal based catalysts, such as oxides,^2–5^ hydroxides,^6–8^ carbides,^9–11^ nitrides,^12,13^ and phosphides^14,15^ have been explored as technologically viable alternatives. Recently, molybdenum carbides, in particular Mo_2_C and MoC, have attracted widespread attention as promising HER catalysts in both acidic and alkaline electrolytes, since the d-band electronic structure of Mo is similar to that of the benchmark Pt catalyst.^16–21^ However, the use of Mo-carbides for the OER is far less explored. This is partially due to stability challenges, as these materials can be oxidized under OER conditions, leading to aggregation and corrosion together with significant loss of catalytic activity. One attractive solution to this end is the deposition of Mo-carbides with an early transition metal (*e.g.* Co, Ni, *etc.*) or metal oxide on molybdenum carbides,^22–24^ leading to heterogeneous composites with enhanced activity and stability. For instance, Dong *et al.* reported nitrogen-doped carbon encapsulating γ-MoC/Ni heterostructures (γ-MoC/Ni@NC) as efficient OER electrocatalysts due to the synergistic effect between γ-MoC and Ni, leading to significantly improved OER activity with a low overpotential of 310 mV to afford a current density of 10 mA cm^−2^.^25^

The straightforward method for preparing early transition metal hybridized molybdenum carbides is the one-pot calcination of mixtures of early transition metal containing salts and Mo precursors. However, most traditional syntheses of these materials involve high-temperature pyrolysis, giving little molecular control over the resulting structure of the composites. As a result, the composites often exhibit component agglomeration and non-ideal morphologies. In contrast, for high electrocatalytic performance, the ideal scenario is the maximum dispersion of accessible reactive sites on the electrode surface. To this end, molecular metal oxides or polyoxometalates (POMs) are ideal Mo-carbide precursor candidates as their structure, composition and reactivity can be tuned at the molecular level.^26,27^ POMs have been successfully used as molecular catalysts in electrochemical energy conversion and storage like water electrolysis^4,28–35^ and batteries.^35–40^ In addition, POMs have been employed as molecular precursors for nanostructured metal oxides and carbides as highly efficient electrocatalysts for the water–oxygen redox cycle.^41–43^ However, challenges still remain including the poor mechanical and electrical linkage between POM and the electrode support, resulting in leakage under operation and high electrical resistance, *i.e.* poor catalytic performance.

**Scheme 1:**
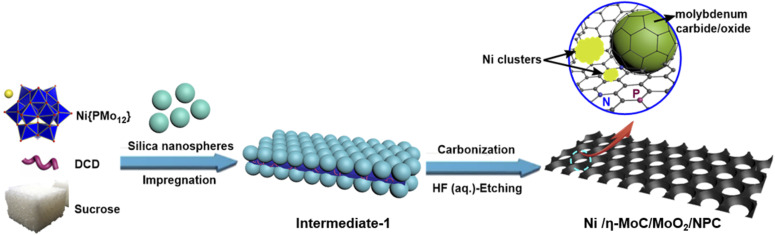
Schematic fabrication approach leading to 2D mesoporous Ni clusters/η-MoC/MoO_2_/NPC.

Herein, we present a novel *in situ* method for depositing highly dispersed Ni metal clusters hybridized with molybdenum carbides/oxides onto high-porosity carbon electrodes. This method utilizes the solution-stable cation-substituted Keggin type polyoxometalate Ni[HPMo^VI^_12_O_40_] (=Ni{PMo_12_}), which combines Brønsted acidity, redox activity, and catalytic reactivity.^44^ Ni{PMo_12_} serves as the single-source molecular precursor for depositing reactive Ni clusters and molybdenum carbides/oxides onto a high surface-area conductive mesoporous carbon electrode.^45–50^ To the best of our knowledge, this is the first example where both the cation and anion of a POM salt contribute to the formation of nanocomposites. The synthetic procedure of the composites is illustrated in Scheme 1. Briefly, the composites were synthesized *via* a hard-templating route, where a commercial mesoporous silica template was impregnated with aqueous solutions containing sucrose as the main carbon precursor and Ni{PMo_12_} as a Brønsted-acidic carbonization catalyst and bimetallic NiMo source.^51^ Dicyandiamide (DCD) served as both the carbon source for the formation of molybdenum carbides and the nitrogen source for N-doping of the carbon matrix. Also, this study shows that the reaction of DCD with Ni{PMo_12_} results in the formation of sheet-like supramolecular structures of intermediate-1 (see Fig. S1).^52,53^ Subsequent carbonization of the intermediate-1 at elevated temperature in an argon atmosphere results in the one-step formation of composites 1 (650 °C), 2 (750 °C) and 3 (850 °C). Notably, composite 2, synthesized at the optimized temperature of 750 °C, is composed of sub-nanometer Ni clusters combined with crystalline η-MoC and MoO_2_ nanoparticles deposited on the N, P-doped mesoporous carbon (Ni clusters/η-MoC/MoO_2_/NPC). Composite 2 showed outstanding electrocatalytic OER activity with a low overpotential at a current density of 10 mA cm^−2^ (*η*_10_ = 320 mV) under alkaline conditions, approaching that of commercial IrO_2_. Mechanistic analyses attribute the enhanced performance to synergistic effects between η-MoC and MoO_2_, with additional benefits from the highly dispersed Ni clusters and N, P co-doping in the carbon matrix. Thus, this work introduces new scalable materials fabrication routes, offering access to high-performance transition metal/metal-oxo clusters embedded in metal carbides/oxides for robust multi-electron electrocatalysis.

## Results and discussion

**Fig. 1:**
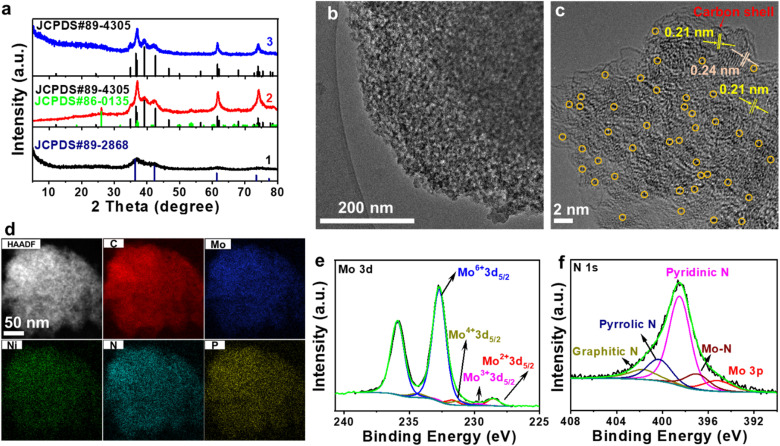
(a) PXRD analysis of composites 1–3. The PDF cards of different chemical phases have been added as indexes: α-MoC_1−*x*_ (JCPDS # 89-2868), η-MoC (JCPDS # 89-4305) and MoO_2_ (JCPDS # 86-0135). (b) AC-TEM and (c) AC-HRTEM images of 2. (d) HAADF-STEM and corresponding EDS mapping of 2, showing the homogeneous dispersion of Mo, Ni, N and P on the mesoporous carbon. (e) and (f) High resolution deconvoluted XPS spectra of Mo 3d and N 1s for 2.

Powder X-ray diffraction (pXRD) analysis (Fig. 1a) shows the different crystalline structures of composites 1–3. Briefly, 1 contains α-MoC_1−*x*_ (JCPDS # 89-2868), 2 contains η-MoC (JCPDS # 89-4305) and MoO_2_ (JCPDS # 86-0135), and 3 contains η-MoC. Note that we do not observe crystalline Ni metal species in any of the samples. We also synthesized three reference samples for comparison: Ref.1 contains α-MoC_1−*x*_, Ref. 2 contains a mixture of MoO_2_ and MoO_3_, and Ref.3 contains a mixture of α-MoC_1−*x*_, Mo_2_C (JCPDS # 72-1683), and Na_5_NiO_4_ (JCPDS # 70-0734) (see details in Fig. S2 and Table S1). The references were synthesized at identical pyrolysis temperatures to 2; significant differences between Ref.1 and Ref.2 were observed. We note that the chemical composition of the samples was dependent on the presence of a carbonization catalyst Ni{PMo_12_}, the presence of DCD and the pyrolysis temperature. Specifically, under our conditions, we note that DCD is critically important for the formation of molybdenum carbide (for details see Table S1). It should be noted that Ref.3 was synthesized using a mixture of NiCl_2_ and Na_2_MoO_4_ in a molar ratio of 1 : 12 rather than the single precursor Ni{PMo_12_}. The pXRD analysis reveals distinctly different results: a large crystalline agglomeration of Na_5_NiO_4_ was observed in the resulting composite. The N_2_ sorption isotherm curves for all samples (1, 2 and 3) displayed a similar type IV isotherm characteristic of micro/mesoporous materials (see Fig. S3a) with the mesoporosity introduced by the silica template. The Brunauer–Emmett–Teller (BET) surface areas for all samples were comparable (between 80 and 110 m^2^ g^−1^, for details see Table S2). The pore size distributions (2–22 nm) of all samples indicated the presence of mesoporous structures (see Fig. S3b).

Next, aberration-corrected high resolution transmission electron microscopy (AC-HRTEM) was performed to examine the morphology of the composites. Fig. 1b and S4 (see the SI) show the large-scale 2D spongy mesoporous carbon structures for 2, indicating the templating effect of both POM and silica. Fig. 1c shows the HRTEM image of 2. Small crystalline nanoparticles with a size of 2–3 nm were embedded into the mesoporous carbon structure. The lattice space of 0.21 nm corresponds to the (104) plane of η-MoC, while that of 0.24 nm corresponds to the (006) plane of η-MoC and/or (200) plane of MoO_2_, which is in agreement with the pXRD analysis. Meanwhile, the carbon shell coated on the nanoparticle can be also observed, illustrating the synthesis of carbon coated metal carbides/oxides. We observe homogeneously distributed particles with diameters of 0.1–0.6 nm doped into the carbon structures, which are attributable to Ni clusters (Ni_*n*_, *n* = 1–5). High angle annular dark-field scanning TEM (HAADF-STEM) imaging together with energy dispersive X-ray spectroscopy (EDS) mapping analysis showed the homogeneous distribution of the elements Mo, Ni, P, and N within the carbon matrix (Fig. 1d), which is in line with the presence of sub-nanometer Ni clusters within the catalyst. Similar morphologies can be also observed for 1 and 3; see details in the SI (Fig. S5–S7). Inductively coupled plasma optical emission spectroscopy (ICP-OES) analyses were performed and revealed Ni loadings of about 1.52 wt% (1), 1.48 wt% (2), and 0.92 wt% (3), respectively (see details in Table S3).

X-ray photoelectron spectroscopy (XPS) was used to assess the elemental composition and chemical species of the catalysts. Survey spectra show the presence of Mo, O, C, and N, while signals for P and Ni were difficult to observe due to their low respective concentration. Composites 1–3 show comparable general XPS features: the Mo 3d spectrum can be deconvoluted into peaks corresponding to Mo^2+^ (attributed to MoC species), Mo^3+^ (attributed to the possible interactions between Mo composites and N), Mo^4+^ (attributed to MoO_2_), and Mo^6+^ (attributed to MoO_3_-based species). MoO_3_ is observed as the predominant species which could be formed by surface oxidation of carbide materials upon exposure of the sample to air.^17,18,54^ The integrated C 1s, N 1s, P 2p, and O 1s analyses confirm the successful doping of N and P into the carbon matrix. For detailed XPS analyses, see Tables S3 and S4 and Fig. S8–S10. Note that previous studies have revealed that catalyst nanoparticles encapsulated in N, P-doped carbon materials can lead to enhanced catalytic activity and high stability in electrocatalysis.^51^ Here, we note that 2 features the highest percentages of Mo^4+^ and Mo^6+^ species, which are attributed to the presence of MoO_2_ in the composite (Fig. 1e and S8–S10, SI). Moreover, pyridinic N is the dominant C–N contribution in all composites, reaching contributions above 69% in 2 (Fig. 1f and S8–S10, SI). The high amount of pyridinic N in 2 might be an important contribution to the electrochemical performance of the composite as high concentrations of pyridinic N improve electrical conductivity and surface wettability of carbon and can facilitate the adsorption of water-oxidation intermediates (OH* and OOH*).^55^

**Fig. 2:**
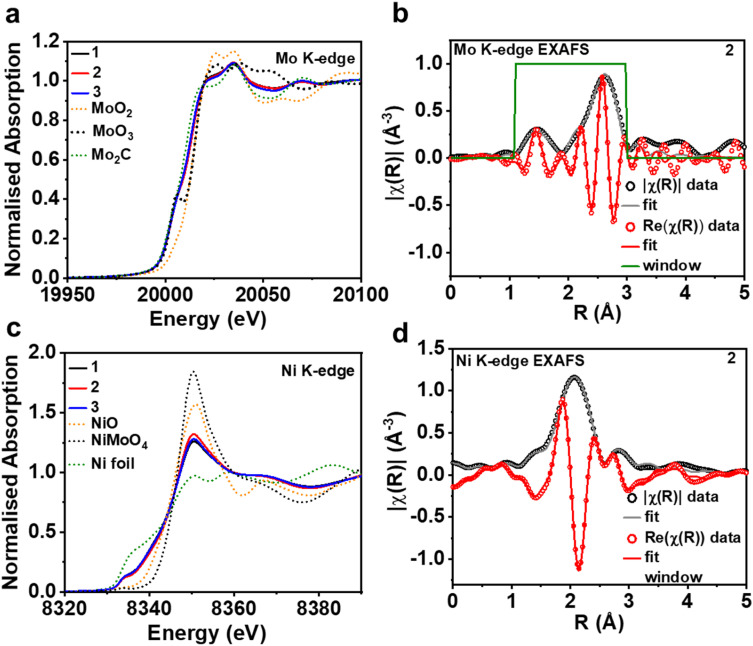
Mo and Ni K-edge XAS data. Stacked plot of the (a) Mo and (c) Ni K-edge XANES data comparing 1–3 with some reference materials. Fitting to the *R*-space of the (b) Mo and (d) Ni K-edge EXAFS data for 2 showing the magnitude (black) and real components of R (red).

To gain further insights into the metal oxidation states and coordination environments in 1–3, we performed X-ray absorption spectroscopy (XAS) analyses at the SuperXAS beamline of the Swiss Light Source (SLS). *Ex situ* XAS experiments were performed at the Mo and Ni K-edges of 1–3, Ref.1, and Ref.2. Furthermore, we used reference materials to probe the oxidation state and local coordination geometry of Mo (using Mo_2_C, MoO_2_, and MoO_3_) and Ni (using Ni foil, NiO, and NiMoO_4_). A stacked plot of the X-ray absorption near edge structure (XANES) data along with fitting to the *R*-space of extended X-ray absorption fine structure (EXAFS) spectroscopy for 1–3 is shown in Fig. 2 and S11–S13. Based on Mo K-edge XANES results, it is evident that the composites 1–3 are very similar in chemical composition with only subtle differences, and represent a linear combination of Mo_2_C, MoO_2_, and MoO_3_ species (Fig. 2a), see Table S5 for the results of XANES linear combination fitting. Note that 2 shows the highest content of Mo-oxides (MoO_2_ and MoO_3_) amongst the catalysts studied, which is in agreement with XPS analyses. Also, Ref.1 shows similar features to those of the composites 1–3, while Ref.2 does not show any contribution from Mo_2_C and is fitted as a linear combination of MoO_2_ and MoO_3_ (Fig. S11a). This agrees with the XRD analysis. The FT of the Mo K-edge EXAFS shows that 1–3 and Ref.1 are dominated by nanocrystalline Mo_2_C species (Fig. 2b and S12, SI), which is in line with the XANES analysis. A full EXAFS analysis was performed to establish the coordination numbers (CN) for the first shell (Mo–C/O) and the second shell (Mo–Mo) interactions and is given in Table S6. Analysis further confirms nanocrystalline molybdenum carbide as the dominant species in these composites.^56^

In Ni K-edge XANES, the three composites 1–3 are also very similar in nature with only subtle differences (Fig. 2c). From visual inspection, these samples appear to be a linear combination of an oxidized and a metallic phase. The samples show an increased intensity as compared to either the NiO or Ni standards between 8360 eV and 8370 eV. Previously, this difference has been attributed to isolated octahedrally coordinated Ni^n+^ species during the incorporation of Ni into perovskite structures.^57–59^ This may suggest that Ni forms small metallic clusters on the outer surface, with a proportion of Ni being incorporated into an octahedral coordination similar to a NiMoO_4_ local structure, which may suggest close interaction between a portion of the metallic Ni clusters and the molybdenum carbide/oxide species. Note that linear combination analysis of the Ni K-edge data is not possible due to the lack of a reference compound for the octahedrally coordinated oxidized component observed. However, XANES analysis using the edge position at the 0.2 edge step suggests that the samples contain approximately 40% metallic Ni^0^ and 60% oxidized Ni^*n*+^ species. Note that the Ni K-edge XANES spectrum of Ref.2 (in which no DCD was added during synthesis) closely resembles that of the NiMoO_4_ reference (Fig. S11b, SI), which exhibits a higher Ni oxidation state compared to samples 1–3. This suggests that DCD plays a crucial role in promoting the formation of small Ni clusters during the calcination process. A full Ni K-edge EXAFS analysis of 1–3 and the reference samples is given in Fig. 2d and S13, SI. Table S6 in the SI shows the fitting results. The above results, in agreement with the XANES data, reveal a structural motif characterized by minimal metallic coordination and partial oxygen coordination.

**Fig. 3:**
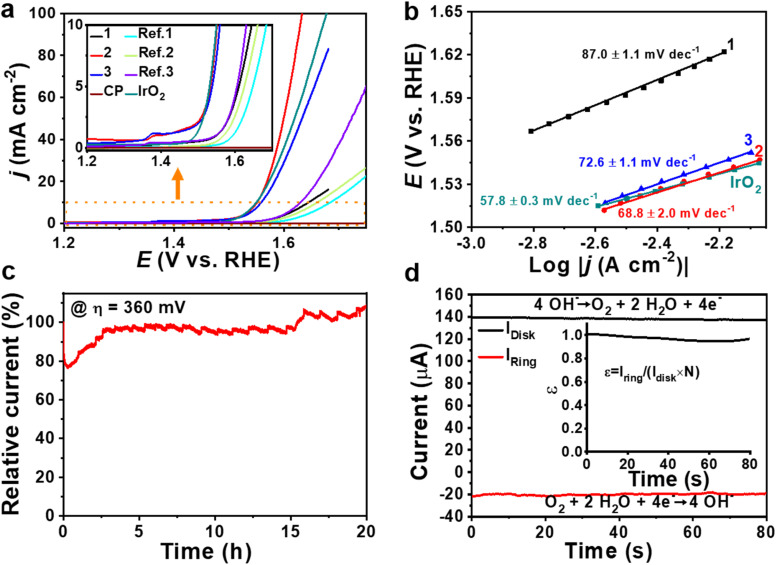
OER study for different catalysts in 1 M KOH. (a) LSV curves. The inset shows the enlarged area shown in the orange box (till *j* = 10 mA cm^−2^). (b) Tafel slopes from LSV curves. (c) Chronoamperometry of 2 at *η* = 360 mV in 1 M aqueous KOH. (d) Evidence of O_2_ generated from 2 using RRDE measurements. The O_2_ gas generated from 2 on the disk (OER current given as *I*_disk_) is reduced at the Pt ring (ORR current given as *I*_ring_, *E*_ring_ = 0.4 V *vs.* RHE, 1600 rpm). The inset is the corresponding faradaic efficiency of 2 for the OER.

The electrocatalytic OER activity of the as-prepared composites 1–3, and other contrast samples was investigated in alkaline solution (1 M KOH) using a standard three-electrode system. The pre-treated carbon paper (CP; see the pre-treatment in the SI) modified with the samples was used as the working electrode (see details in the SI for the procedures of depositing the catalysts on CP). A graphite rod was used as the counter electrode and a Hg/HgO electrode was used as the reference electrode (see details in the Experimental section in the SI). Fig. 3a shows the linear sweep voltammetry (LSV) curves of different samples, while commercial IrO_2_ was studied as the reference catalyst. For composites 1–3, a pre-catalytic oxidation peak was observed at ∼1.4 V, which is attributed to the oxidation of Ni^2+^ to Ni^3+^, a process that has been observed for most Ni-based electrocatalysts.^25,60^ The overpotential at a current density of 10 mA cm^−2^ (*η*_10_) was used to compare reactivity between the catalysts tested. Here, we note that 2 shows the highest electrocatalytic OER activity with the lowest *η*_10_ amongst all the as-prepared catalysts (320 mV, for details see the inset in Fig. 3a and Table 1), which can compete well with commercial IrO_2_ (*η*_10_ = 320 mV). Notably, we observe that the overpotential differences between 2 and others increase with the current density increasing from 10 mA cm^−2^ (Fig. 3a). Compared with 3, we observe significantly better performance by 2, indicating that the presence of both η-MoC and MoO_2_ is critical for the OER activity of 2. The results indicate that the catalytic activity is governed by the distinct chemical species present in composites 1–3, which were synthesized at different pyrolysis temperatures.

**Table 1:** Comparison of the electrocatalytic OER performance of different catalysts

Composite	*η* (mV) @ 10 mA cm^−2^	Tafel slopes (mV dec^−1^)	ECSA (cm^2^)	*R*_ct_ (Ohm)
1	410	87.0 ± 1.1	217.3 ± 0.8	2.4
2	320	68.8 ± 2.0	254.3 ± 1.5	1.2
3	330	72.6 ± 1.1	201.8 ± 1.0	3.2
Ref.1	450	—	—	—
Ref.2	430	—	—	—
Ref.3	400	—	—	—
IrO_2_	320	57.8 ± 0.3	—	—

Based on LSV data, we performed Tafel slope analysis to quantitatively compare reaction kinetics for the catalysts. As shown in Fig. 3b, in the low overpotential range, 2 exhibited the lowest Tafel slope (68.8 ± 2.0 mV dec^−1^) amongst all the as-prepared catalysts, which (under the given conditions) is approaching the Tafel slope of IrO_2_ (for details see Table 1). Note that 2 also compares favourably with related literature-known molybdenum carbide-based catalysts (see Table S7). In addition to reactivity, high stability under OER conditions is a second critical parameter for technological deployment. Chronoamperometric (CA) tests (Fig. 3c) show that 2 exhibits excellent durability in alkaline electrolyte. After an initial drop (which is common for this type of material),^61^ the current gradually increases and stabilizes at ∼105% of its initial value after 20 h, suggesting a mild activation of the catalyst.

To investigate the potential chemical change during catalysis, we performed *ex situ* XAS measurements for 2 at different CA operation times. Linear combination fitting of Ni K-edge XANES using Ni and NiO shows the increasing amount of NiO and the decreasing amount of metallic Ni during this study (see Fig. S14). Based on the literature, the *in situ* partial oxidation of Ni species could be linked to increasing OER activity.^62,63^ Furthermore, post-catalytic analyses of 2 were performed after the CA test. TEM and EDS mapping data show that the morphology of the catalyst and distribution of the elements do not change during electrocatalysis (see Fig. S15). Nevertheless, we observe the formation of MoO_3_ after OER catalysis, indicating the partial oxidation of η-MoC and MoO_2_.^64^ Furthermore, the Mo 3d XPS spectrum (see Fig. S16) also shows partial oxidation of the low-valent Mo^2+^ and Mo^3+^ species. Although the composites can undergo partial oxidation during the OER process, this does not compromise their OER activity under the given reaction conditions.

The high activity of 2 was linked to the following considerations: (i) the presence of sub-nanometer Ni clusters is essential for the OER;^61,65^ (ii) the combined presence of η-MoC and MoO_2_ seems to result in synergistic OER activity. Note that MoO_2_ nanostructures have previously been reported as promising OER catalysts.^64^ Furthermore, Mo oxides have also been reported as promoters, which enhance the catalytic activity of the host catalyst. For example, Xie *et al.*^66^ reported that adding Mo oxides to the NiFe alloy results in improved electrical conductivity and, consequently, enhanced OER performance. (iii) Co-doping of the carbon matrix with N and P atoms has been reported to enhance the electrical conductivity and OER performance.^67^

To confirm that the observed current in Fig. 3a originates from the OER, we performed a rotating ring-disk electrode (RRDE) measurement for 2 with a ring potential of 0.40 V to reduce the generated O_2_ from the disk electrode, showing a continuous OER (disk electrode) to oxygen reduction reaction (ORR, ring electrode) process. As shown in Fig. 3d, with the disk current held constant at 140 µA (black curve), O_2_ molecules generated from the disk electrode sweep across the surrounding Pt ring electrode and are rapidly reduced at an ORR potential. Consequently, a ring current of ≈20 µA is detected (red curve), verifying that the oxidation current observed in the disk electrode can be fully attributed to the OER with a faradaic efficiency higher than 95%.

The electrochemically active surface area (ECSA) of the composites and the charge transfer resistance (*R*_ct_) at the electrode/electrolyte interface are two main parameters for the reactivity of heterogeneous electrocatalysts, which provide initial mechanistic insights into the superior electrocatalytic performance. Thus, we first performed an electrochemical double-layer capacitance (*C*_dl_, Fig. S17) test to obtain the ECSA values of 1–3.^68,69^ As summarized in Table 1, we observe a moderately increased ECSA for 2 compared with the other composites. Also, electrochemical impedance spectroscopy (EIS) analysis shows that 2 features the lowest *R*_ct_ (Fig. S18 and Table 1), which facilitates electron transfer at the solid-electrolyte interface.

## Conclusions

In conclusion, we report a novel POM/silica-templated 2D mesoporous N, P-doped carbon supported Ni clusters/η-MoC/MoO_2_ composite electrocatalyst for water oxidation in alkaline medium. Low overpotentials, high faradaic efficiencies and long operational times without loss of reactivity are observed. By considering the structural and compositional diversity of POMs, we propose that this new materials design concept enables the targeted development of complex multicomponent composite catalysts and their simultaneous electrical wiring to mesoporous carbon structures. The study could therefore provide a protocol for the development of novel (mixed)metal carbides/oxides as (electro-)catalysts for challenging multielectron transfer reactions.

## Author contributions

Rongji Liu: conceptualization, investigation, supervision, formal analysis, writing – original draft, writing – revision; Yupeng Zhao: investigation, formal analysis, writing – original draft; Archismita Misra: investigation, validation; Dandan Gao: investigation, formal analysis, supervision; Adam H. Clark: investigation, formal analysis, methodology; Montaha Anjass: investigation, supervision; Johannes Biskupek: investigation, formal analysis, methodology; Ute Kaiser: investigation, validation, supervision; Guangjin Zhang: resources, supervision, investigation; Carsten Streb: conceptualization, resources, supervision, funding, writing – original draft, writing – revision.

## Conflicts of interest

There are no conflicts to declare.

## Supplementary Material

SC-OLF-D6SC02532C-s001

## Data Availability

The data that support the findings of this study are openly available at https://Zenodo.org at https://doi.org/10.5281/zenodo.21456140. Supplementary information (SI): synthetic, analytical and catalytic data. See DOI: https://doi.org/10.1039/d6sc02532c.
