# Supplementary material for: Sustained oxygen evolution reaction by 2D mesoporous polyoxometalate-derived composite electrocatalysts
Source: Chem Sci. 2026 Jul 16. Online ahead of print. doi: 10.1039/d6sc02532c (PMC13403943; doi:10.1039/d6sc02532c)
Supplement: SC-OLF-D6SC02532C-s001 [file SC-OLF-D6SC02532C-s001.pdf]

Supplementary Information (SI) for Chemical Science.
This journal is © The Royal Society of Chemistry 2026

Electronic Supplementary Information

Sustained oxygen evolution reaction by 2D mesoporous polyoxometalate-derived composite electrocatalysts

Rongji Liu,*[abc] Yupeng Zhao,[ab] Archismita Misra,[b,h] Dandan Gao,*[ab] Adam H. Clark,[d] Montaha Anjass,[b,e] Johannes Biskupek,[f] Ute Kaiser,[f] Guangjin Zhang,[g] and Carsten Streb*[abc]

[a.] *Johannes Gutenberg University Mainz, Department of Chemistry, Duesbergweg 10–14, 55128 Mainz, Germany. E-mails: rongji.liu@uni-mainz.de; dandan.gao@uni-mainz.de, carsten.streb@uni-mainz.de*
[b.] *Institute of Inorganic Chemistry I, Ulm University, Albert-Einstein-Allee 11, 89081 Ulm, Germany.*
[c.] *Helmholtz-Institute Ulm, Electrochemical Energy Conversion, Helmholtzstr. 11, 89081 Ulm, Germany.*
[d.] *Paul Scherrer Institut, Forschungsstrasse 111, Villigen, CH-5232, Switzerland.*
[e.] *University of Sharjah, Department of Chemistry, PO Box 27272 Sharjah, United Arab Emirates.*
[f.] *Central Facility of Electron Microscopy for Materials Science, Ulm University, Albert-Einstein-Allee 11, 89081 Ulm, Germany.*
[g.] *CAS Key Laboratory of Green Process and Engineering, Institute of Process Engineering, Chinese Academy of Sciences, 100190, Beijing, China.*
[h.] *Bundesanstalt für Materialforschung und -prüfung (BAM), Department of Materials and the Environment, Unter den Eichen 87, 12205 Berlin, Germany.*

1. Materials and Methods
2. Synthetic Section
3. Electrocatalysis
4. Supplementary Data
5. Supplementary References

1. Materials and Methods

Materials

All commercial chemicals were used without purification. Phosphomolybdic acid hydrate ($[H_3PMo_{12}O_{40}] \cdot xH_2O$ (= PMo_{12}), Alfa Aesar), sucrose ($C_{12}H_{22}O_{11}$, Merck Millipore (Calbiochem)), silica gel powder (SiO_2, pore size of 150 Å, Sigma-Aldrich), hydrofluoric acid (aqueous HF, ≥ 48%, Sigma-Aldrich), dicyandiamide (DCD, $C_2H_4N_4$, 99%, Sigma-Aldrich), Nickel chloride ($NiCl_2$, Acros Organics), Anhydrous sodium molybdate (Na_2MoO_4, Alfa Aesar). $Ni[HPMo_{12}O_{40}]$ (= Ni{PMo_{12}}) was prepared based on a literature procedure.[1] Distilled water was used throughout the experiments.

Methods

Transmission electron microscopy (TEM) measurements were performed using an image-side aberration corrected FEI Titan 80-300 at 80 kV accelerating voltage for acquistion of AC-HRTEM images. High angle annuar dark-field (HAADF) scanning (S) TEM together with energy dispersive X-ray spectroscopy (EDS) was performed using a Thermofisher *Talos 200X at 200 kV voltage. The system was equipped with a windowless 4-quadrant EDS-detector (SuperX).*

Powder X-ray diffraction (pXRD) was performed on a BRUKER D8 Advance XRD unit using Cu−Kα (λ = 1.54 Å).

X-ray photoelectron spectroscopy (XPS) analysis was performed on ESCALAB250 Thermo Electron Corporation equipment with an Al Ka X-ray source (1486.6 eV). The X-ray source was run at a reduced power of 150 W, and the pressure in the analysis chamber was maintained at <10^{-11} Pa.

Inductively coupled plasma optical emission spectroscopy (ICP-OES) was performed on a Perkin Elmer Plasma 400 spectrometer. CV and LSV experiments were performed on a CHI 730E electrochemical workstation (CH Instruments Inc.).

Nitrogen Physisorption: Sample degassing was performed on a Micromeritics Smart VacPrep at 100 °C for 12h. Nitrogen sorption isotherms were acquired on a Micromeritics 3Flex at T = 77 K and evaluated using Micromeritics 3Flex Software Version 5.00. N_2 was used as adsorbate. Calculation of the BET surface area was accomplished by the

bet_autofit_v0712 method from Micromeritics. The evaluation of the pore distribution after BJH was done by using Harkins and Jura thickness curve and the Kruk-Jaroniec-Sayari correction. The evaluation of the micropores was realized by use of a adapted Harwarth-Kawazoe method estimating a cylindrical pore geometry after Saito-Foley.

Electrochemical impedance spectroscopy (EIS) experiments were performed on a CHI 760E electrochemical system (CH Instruments Inc.) in the frequency range from 1000 kHz to 0.01 Hz with modulation amplitude of 5 mV.

X-ray absorption spectroscopy (XAS) analysis: Ex-situ Mo K-edge XAS experiments were carried out at the SuperXAS beamline of the Swiss Light Source.[2] The storage ring operated at 2.4 GeV in top-up mode with a ring current of 400 mA. The polychromatic X-ray beam resulting from a 2.9 Tesla bending magnet was collimated by a Pt-coated mirror at 2.5 mrad (which also served to reduce higher-order harmonics) and subsequently monochromatised by a Si (111) channel-cut monochromator. A Rh coated toroidal mirror was used to refocus the beam onto the sample spot. Data were collected in transmission geometry using the quick-scanning extended X-ray absorption fine-structure spectroscopy (QEXAFS) mode at 1 Hz monochromator oscillation frequency. Ionization chambers 15 cm long were filled with 1 bar N_2-filled at the Ni K edge and 0.5 bar Ar, 0.5 bar N_2 filled at the Mo K edge. A Mo and Ni reference foils respectively were mounted between the second and third ionization chambers was measured simultaneously for absolute energy calibration. The samples were prepared as 13 mm pellets with the MoO_2, MoO_3 and Mo_2C formed with approximately 20 mg diluted with 40 mg of cellulose and Samples **1**, **2**, **3** formed with approximately 40 mg diluted with approximately 20 mg of cellulose. The QEXAFS spectra were processed using ProQEXAFS to extract individual XAS spectra, calibrate, normalise and average 5 min of data.[3]

2. Synthetic Section

Preparation of Ni[$HPMo_{12}O_{40}$] derived composites: 1.318 g (0.7 mmol) of Ni{PMo_{12}} was dissolved in 2.5 mL of water before 0.625g (1.81 mmol) of sucrose was added and dissolved. Finally, 0.5 g of commercial silica gel powder was dispersed in the above solution and stirred overnight at room temperature. The mixture was heated in air to 100 °C for 6 h and subsequently to 160 °C for another 6 h to evaporate the water. This impregnation process was repeated with a second solution containing 0.4 g (1.16 mmol) of sucrose and 0.847 g (0.45 mmol) of Ni{PMo_{12}} in 2.5 g of water. To the above air-dried materials, 15 ml water containing 4.33 g DCD was added and stirred at 300 rpm for 5 h. The mixture was then heated at 100 °C for 6 h in the air, leading to the dried 2D supramolecular nanoscale structure named **intermediate-1**. At last, the **intermediate-1** was carbonized at different temperatures (650, 750 and 850 °C) for 5 h under Ar at a heating rate of 5 °C min^{-1}. To remove the silica template, the as-prepared composites were stirred in 30 ml of 10% aqueous HF for 48 h. **Attention, extreme care and expertise is required when handling hydrofluoric acid as it is highly poisonous and can be absorbed through the skin.** The composites were then washed with water (3x) and ethanol (3x) and dried at 100 °C overnight, resulting in composites **1** (650 °C), **2** (750 °C) and **3** (850 °C).

Preparation of reference samples: For comparison, PMo_{12} was also used as a carbonization catalyst instead of Ni{PMo_{12}}, and the synthetic procedure was kept the same as **2**, resulting in reference 1 (**Ref.1**). Reference 2 (**Ref.2**) was prepared identically to **2** without addition of DCD. Reference 3 (**Ref.3**) was prepared identically to **2** while using a mixture of $NiCl_2$ (0.7 mmol) and Na_2MoO_4 (8.4 mmol) in a molar ratio of 1:12, rather than the single precursor Ni{PMo_{12}}. All the other conditions were kept the same as **2**.

3. Electrocatalysis

5 mg of the finely ground catalysts (**1**, **2**, **3**, **Ref.1**, **Ref.2**, **Ref.3** or **IrO_2**) was dispersed in 960 µL iso-propanol containing 20 µL water and 20 µL 5% Nafion solution ([catalyst] = 5 mg mL^{-1}) by sonication for 1 h to form a homogeneous ink. The carbon paper (0.5 × 10 × 30 mm) was used as the catalyst support, which was washed and sonicated in acetone, ethanol and water for 20 min each before use. Then, 20 µL of the catalyst ink were separately dropped onto pre-treated carbon papers with a fixed geometric surface area of 0.2 cm^2 (the loading amount was 0.5 mg cm^{-2}). After drying, the electrodes were further modified with a thin film of Nafion by dropping 5 µL 0.5 wt% Nafion solution (in iso-propanol) onto the catalyst surface. A standard three-electrode cell was used and was operated at room temperature. For OER studies, the prepared thin-film covered carbon paper was used as the working electrode, and the graphite rod and an Hg/HgO (1 M KOH) electrode were used as counter electrode and reference electrode, respectively. The electrode was performed 50 CV (0 to 1 V vs. reversible hydrogen electrode (RHE), scan rate: 0.1 V s^{-1}) cycling beforehand for stabilization. OER measurements were performed on a CHI 730E electrochemical system (CH Instruments Inc.). The scan rate for LSV measurements was 0.005 V s^{-1}. EIS experiments were performed in the same electrolyte on a CHI 760E electrochemical system (CH Instruments Inc.) in the frequency range from 1000 kHz to 0.01 Hz with modulation amplitude of 5 mV. For Faradaic efficiency studies, 7.5 µL of the catalyst ink were loaded onto a glassy carbon (GC) rotating ring-disk electrode (RRDE) with 4 mm diameter (the loading of the catalysts was 0.3 mg cm^{-2}) and used as working electrode.

The Hg/HgO electrode was referenced against the RHE in all measurements. The referencing was performed based on the Nernst equation:

$E_{RHE} = E_{Hg/HgO} + E^0_{Hg/HgO} + 0.059$ pH.

For 1 M aqueous KOH (pH = 13.7): $E_{RHE} = E_{Hg/HgO} + 0.927$ V.

ECSA values are calculated based on the equations shown below:

(1) $\Delta j = \gamma \times C_{dl}$

(2) ECSA = C_{dl}/C_s

(3) $C_s = 40\ \mu F/cm^2$

where γ is the scanning rate, Δj (= j_a - j_c) is the charging current density differences, C_{dl} is the double-layer capacitance and C_s is the specific capacitance of the catalyst.

4. Supplementary Data

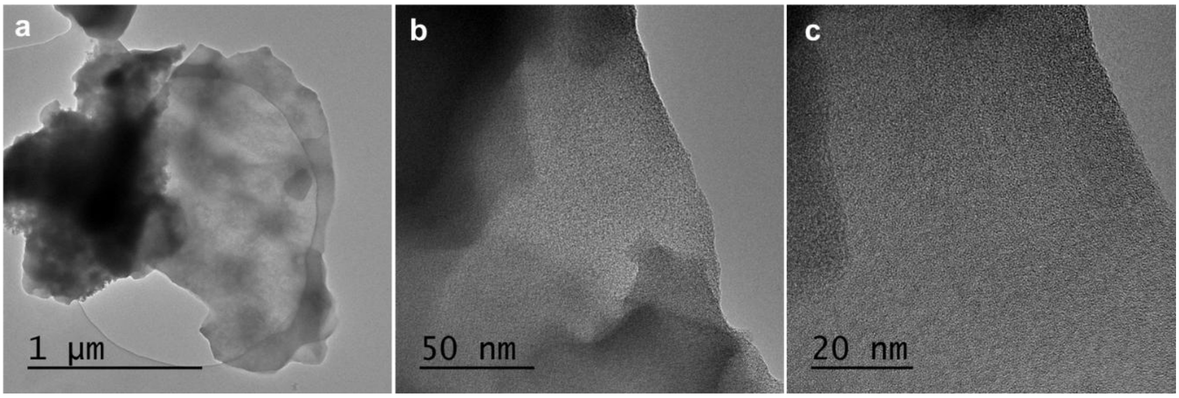

Fig. S1. Overview TEM image (a) and AC-HRTEM images (b,c) of the synthesized **intermediate-1**, indicating the 2D sheet-like structure due to the electrostatic interaction between Ni{PMo_{12}} and DCD.

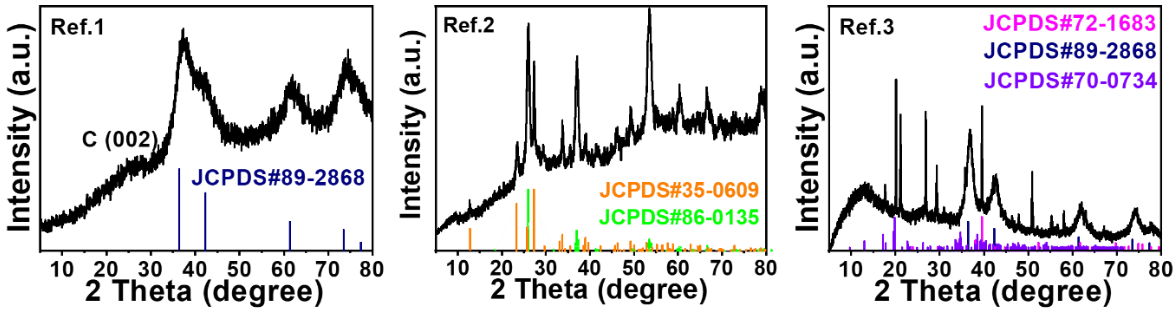

Fig. S2. Powder X-ray diffraction patterns for **Ref.1**, **Ref.2, and Ref.3**. The vertical lines show the PDF cards of α-MoC_{1-x} (JCPDS # 89-2868), MoO_2 (JCPDS # 86-0135), MoO_3 (JCPDS # 35-0609), Mo_2C (JCPDS # 72-1683), and Na_5NiO_4 (JCPDS # 70-0734).

Table S1. Crystalline components (based on pXRD analysis) and synthetic conditions for the different catalysts

**Samples**	**Composition**	**Pyrolysis temperature (˚C)**
**1**	α-MoC_{1-x}	650
**2**	η-MoC and MoO_2	750
**3**	η-MoC	850
**Ref.1**	α-MoC_{1-x}	750
**Ref.2**	MoO_2 and MoO_3	750
**Ref.3**	α-MoC_{1-x}, Mo_2C and Na_5NiO_4	750

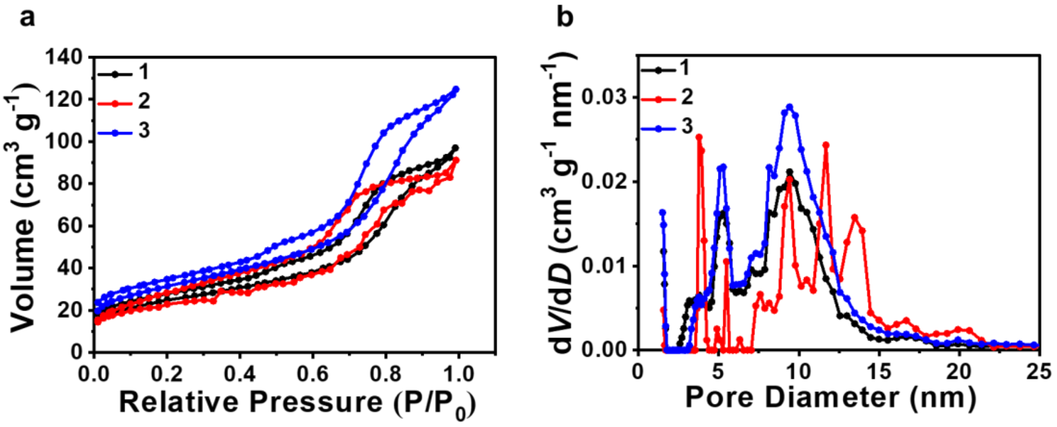

Fig. S3. (a) Nitrogen sorption isotherms and (b) the corresponding pore size distributions of **1**, **2** and **3**.

Table S2 Nitrogen sorption analyses for different materials.

**Samples**	**BET specific surface area (m^2/g)**	**Pore volume (cm^3/g)**	**Pore diameter（nm）**
**1**	87.797	0.139	9.416
**2**	79.619	0.119	3.775
**3**	111.308	0.182	9.416

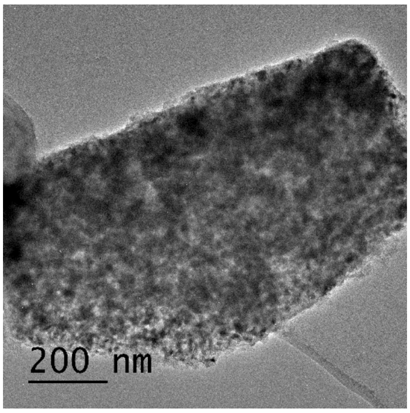

Fig. S4. Overview TEM image of **2**, showing the large scale 2D spongy mesoporous carbon structures.

Table S3. Atomic contents of different elements in different samples

**Samples**	**Atomic content (at. %)[a]**						**Weight content (wt. %)[b]**	
	N	C	O	P	Mo	Ni	Mo	Ni
**1**	3.06	75.0	17.7	0.26	3.91	0.10	40.12 ± 0.07	1.52 ± 0.21
**2**	2.59	73.4	19.4	0.27	4.15	0.12	34.16 ± 0.10	1.48 ± 0.15
**3**	1.08	74.3	18.2	0.33	5.92	0.16	32.32 ± 0.32	0.92 ± 0.16

[a] The values were determined by XPS analysis
[b] The values were determined by ICP-OES analysis

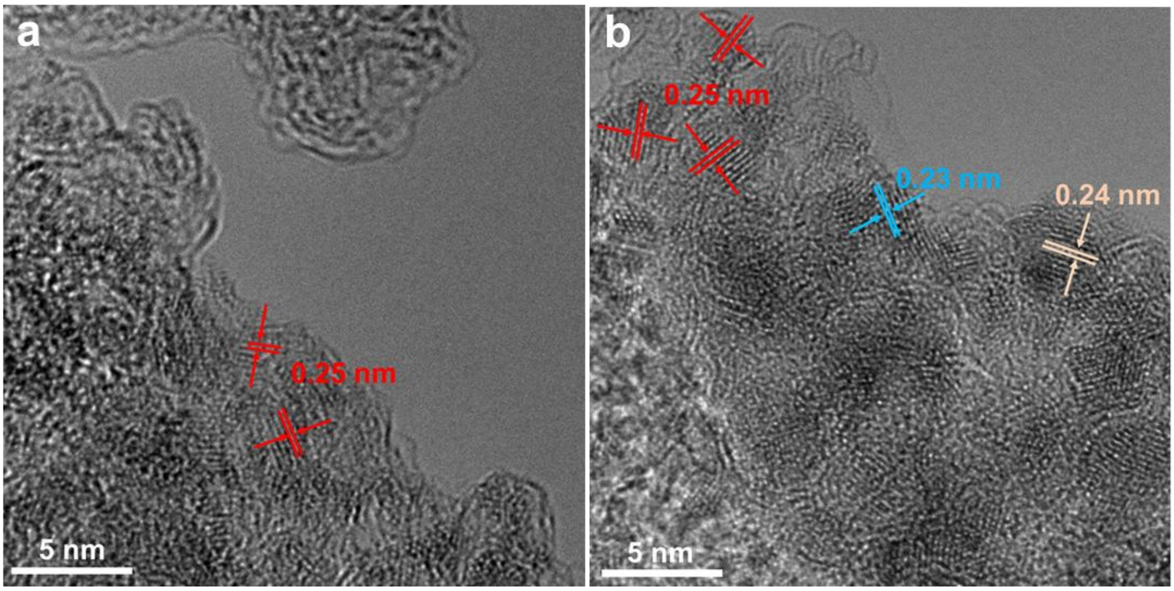

Fig. S5. Aberration-corrected HRTEM images of (a) **1** and (b) **3** showing the presence of crystalline nanoparticles within graphitic carbon support.

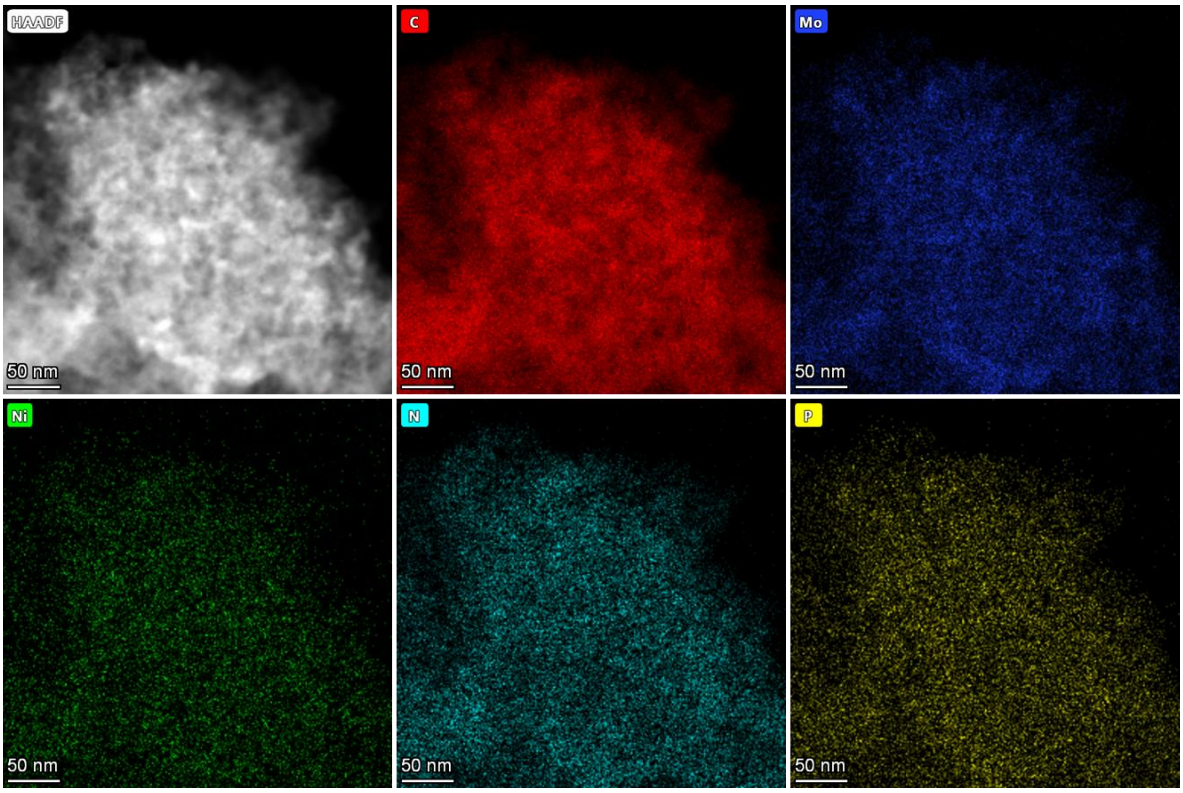

Fig. S6. HAADF-STEM and corresponding EDS mapping of **1**.

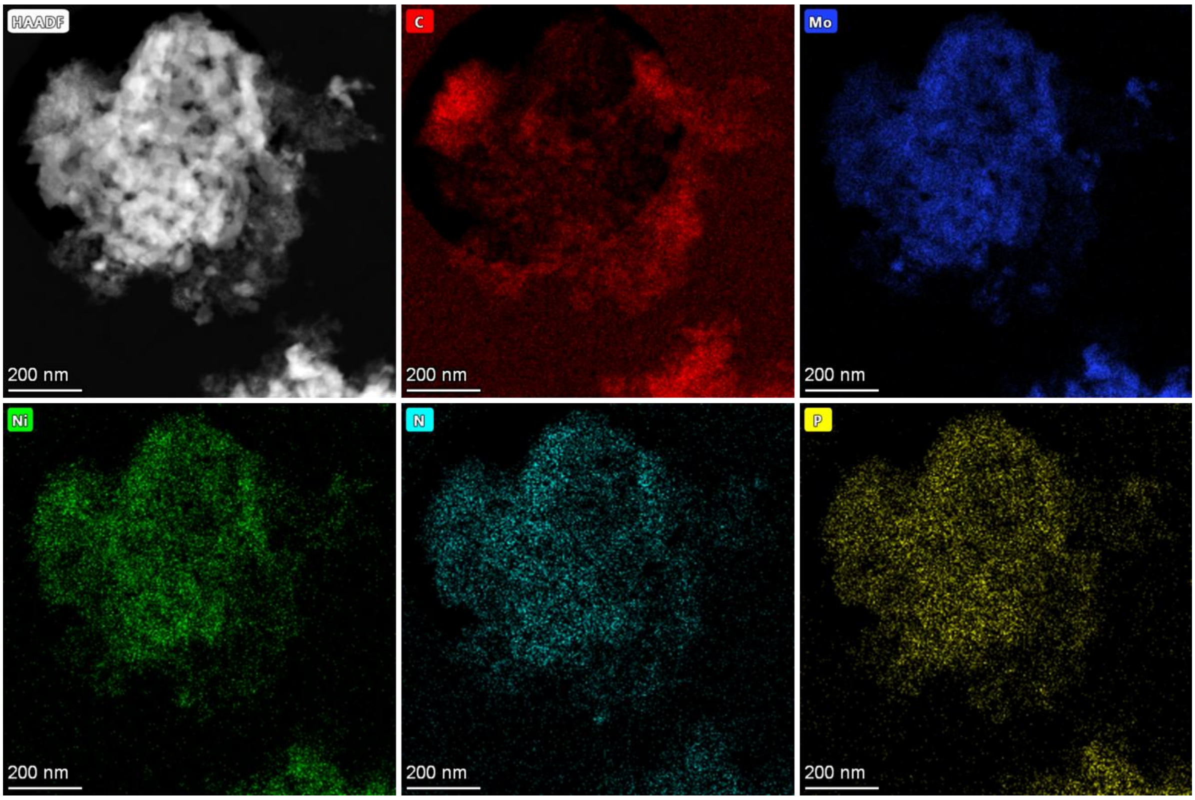

Fig. S7. HAADF-STEM and corresponding EDS mapping of **3**.

Fig. S5 shows the HRTEM images of the different composites**.** For both composites, small crystalline nanoparticles (1-3 nm, details see below) were imbedded into the mesoporous carbon structure with high dispersion. Also, small Ni clusters can be observed in all the samples. Energy dispersive x-ray spectroscopy (EDS) mapping analysis with HAADF-STEM showed the homogeneous distribution of the elements Ni, Mo, P and N within the carbon matrix (Fig. S6-S7). In composite **1**, the lattice space of 0.25 nm corresponds to the (111) plane of α-MoC_{1-x}. In composite **3**, the lattice spaces of 0.23 nm, 0.24 nm and 0.25 nm correspond to the (103), (006) and (102) planes of η-MoC, respectively. These results agree well with the pXRD data.

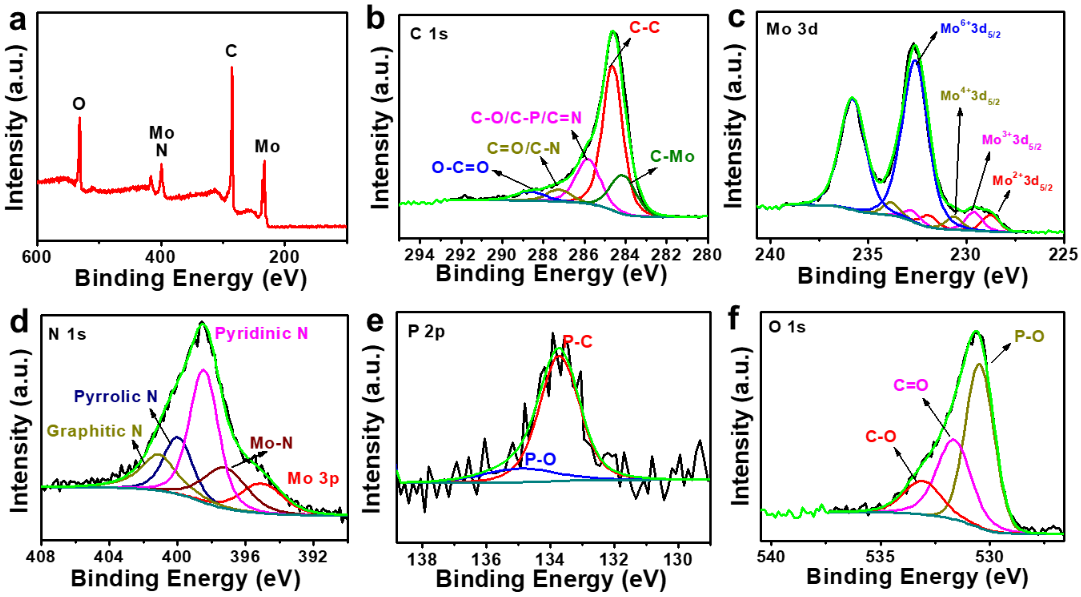

Fig. S8. (a) Survey XPS spectrum of **1**. (b - f) High resolution deconvoluted XPS spectra for **1**, showing C 1s, Mo 3d, N 1s, P 2p and O 1s.

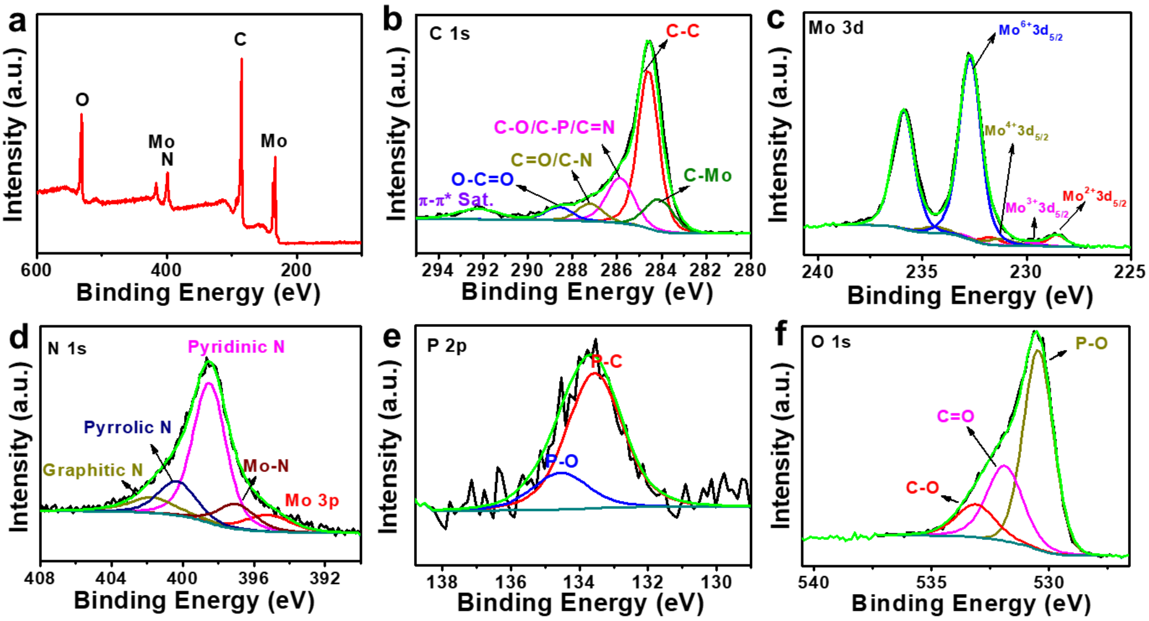

Fig. S9. (a) Survey XPS spectrum of **2**. (b - f) High resolution deconvoluted XPS spectra for **2**, showing C 1s, Mo 3d, N 1s, P 2p and O 1s.

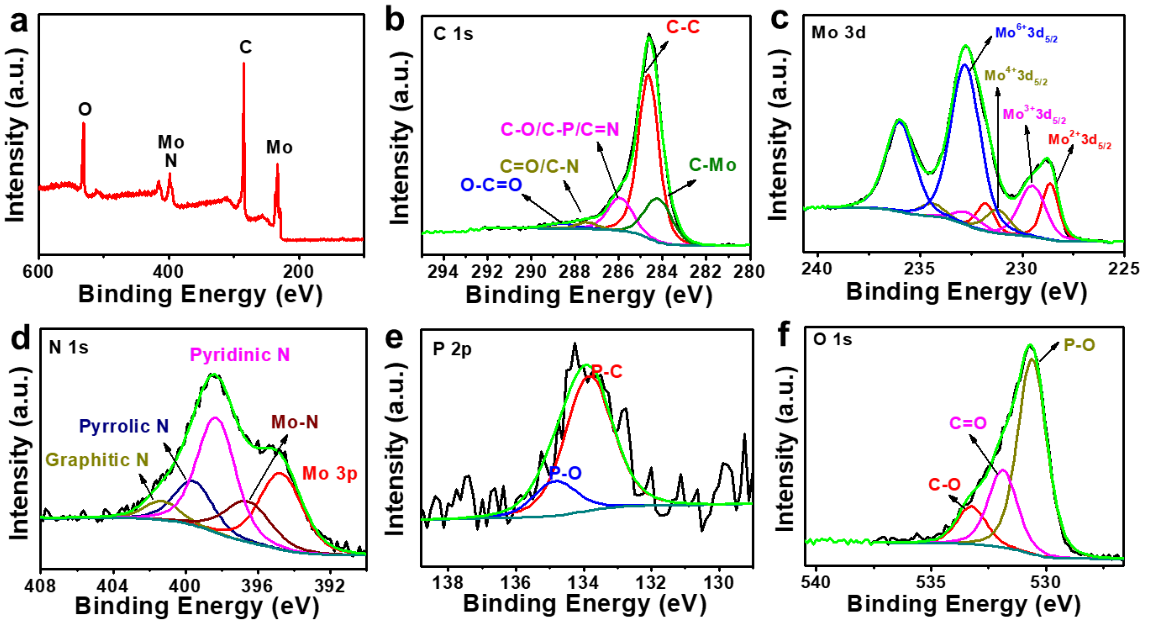

Fig. S10. (a) Survey XPS spectrum of **3**. (b - f) High resolution deconvoluted XPS spectra for **3**, showing C 1s, Mo 3d, N 1s, P 2p and O 1s.

Fig. S8 - S10 show the XPS analyses for composites **1**, **2** and **3**. For all samples, the Mo 3d spectra can be deconvoluted into peaks corresponding to Mo^{2+}, Mo^{3+}, Mo^{4+} and Mo^{6+} (see more details in the main text). The presence of Mo^{4+} and Mo^{6+} can be assigned to Mo oxides formed by surface oxidation of carbide materials which is known for these materials when exposed to air.[4–6] The C 1s spectra exhibited five peaks assigned to C-Mo, C-C, C-O/C-P/C=N, C=O/C-N and O-C=O. The N 1s spectra exhibited four different peaks of Mo−N, pyridinic N, pyrrolic N and graphitic N. Two peaks assigned to P-C and P-O bonds are observed in the P 2p spectra. For the O 1s spectra, three functional groups of P-O, C=O and C-O can be seen. The integrated C 1s, N 1s, P 2p and O 1s analyses conclude the successful doping of N and P in the carbon. For detailed XPS data, see Table S4.

Table S4. Binding energies of different elements from XPS analysis.

Samples		Composite 1	Composite 2	Composite 3
**Mo 3d**	Mo^{II} 3d $_{5/2}$	228.7	228.5	228.6
	Mo^{II} 3d $_{3/2}$	231.9	231.7	231.8
	Mo^{III} 3d $_{5/2}$	229.6	229.7	229.5
	Mo^{III} 3d $_{3/2}$	232.8	232.9	232.7
	Mo^{IV} 3d $_{5/2}$	230.6	231.2	231.2
	Mo^{IV} 3d $_{3/2}$	233.8	234.4	234.4
	Mo^{VI} 3d $_{5/2}$	232.6	232.7	232.8
	Mo^{VI} 3d $_{3/2}$	235.8	235.9	236.0
**P 2p**	P-C	133.7	133.6	133.8
	P-O	134.9	134.6	134.8
**C 1s**	C-Mo	284.1	284.1	284.2
	C-C	284.6	284.6	284.7
	C-O/C-P (/C=N)	285.8	285.9	286.0
	C=O (/C-N)	287.2	287.2	287.5
	O-C=O	288.6	288.6	288.7
**O 1s**	P-O	530.5	530.5	530.6
	C=O	531.6	531.9	531.9
	C-O	533.1	533.1	533.2
**N 1s**	Pyridinic N	398.4	398.5	398.3
	Pyrrolic N	400.0	400.3	399.6
	Graphitic N	401.1	401.7	401.3
	Mo-N	397.2	397.0	396.6

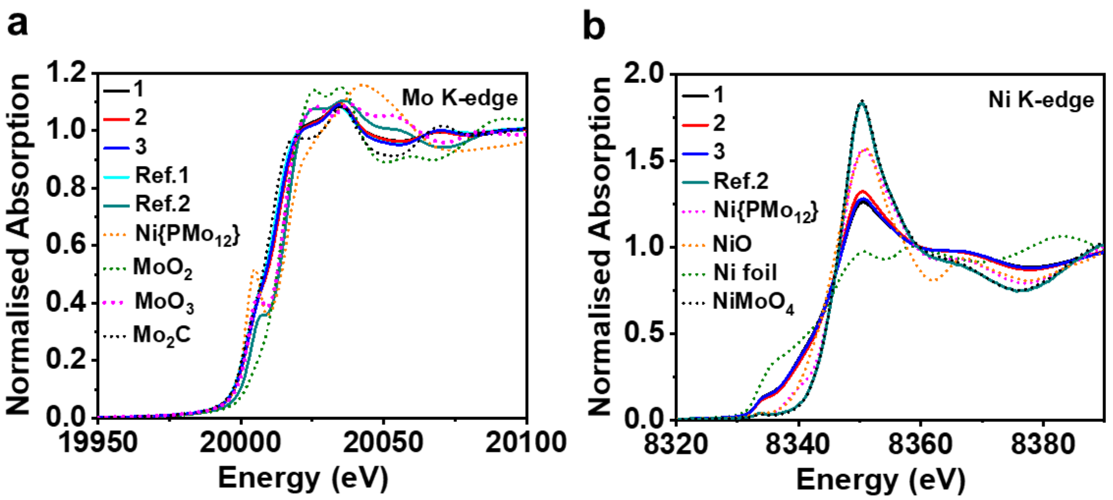

Fig. S11. Stacked plot of the Mo and Ni K-edge XANES data comparing the three samples (**1**, **2** and **3**) with some references. The Mo and Ni K-edge XANES for Ni{PMo_{12}} confirm the valence of Mo^{6+} and Ni^{2+} in the compound.

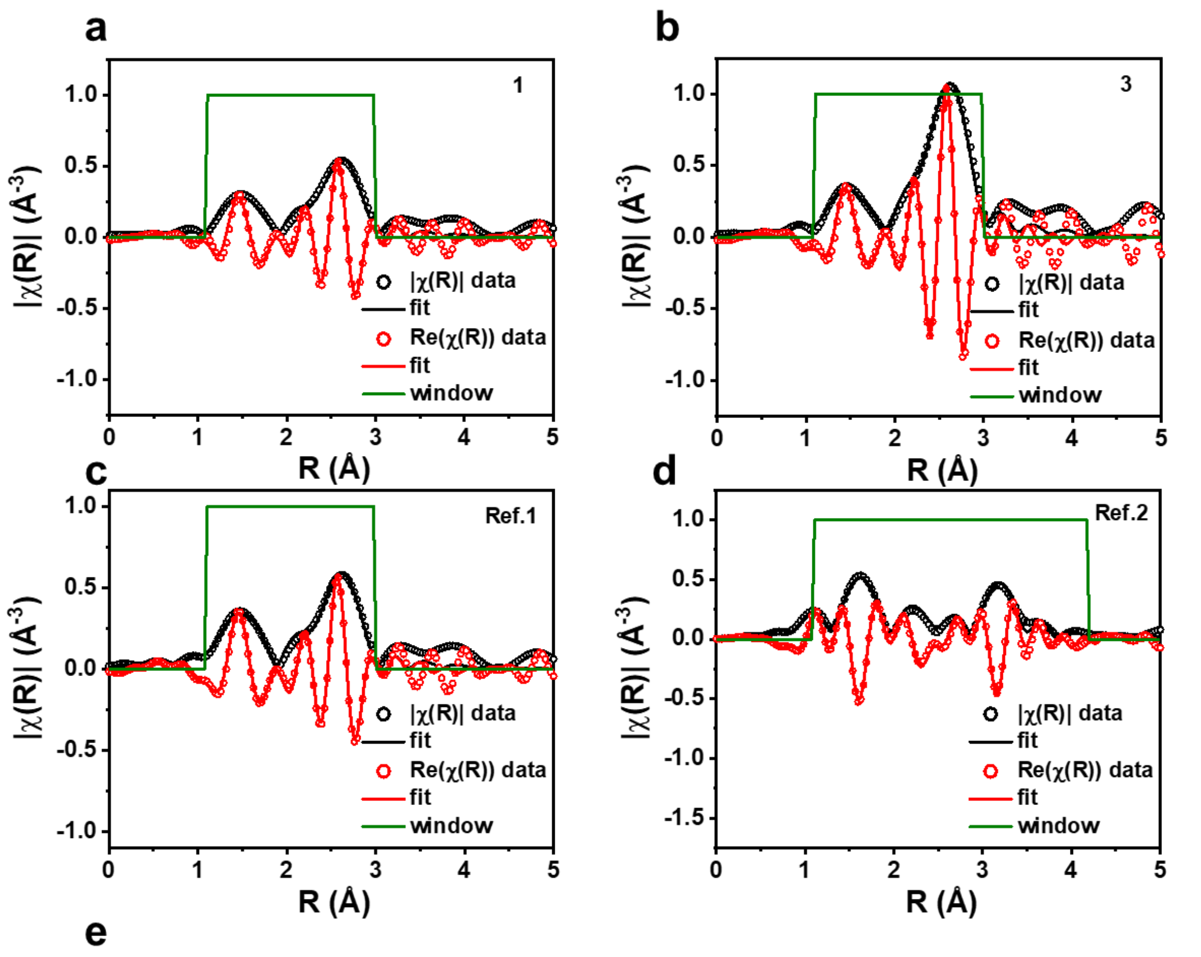

Fig. S12. Fitting to the R-space of the Mo K-edge EXAFS data for composites **1, 3, Ref.1** and **Ref.2** showing the magnitude (black) and real components of R (red).

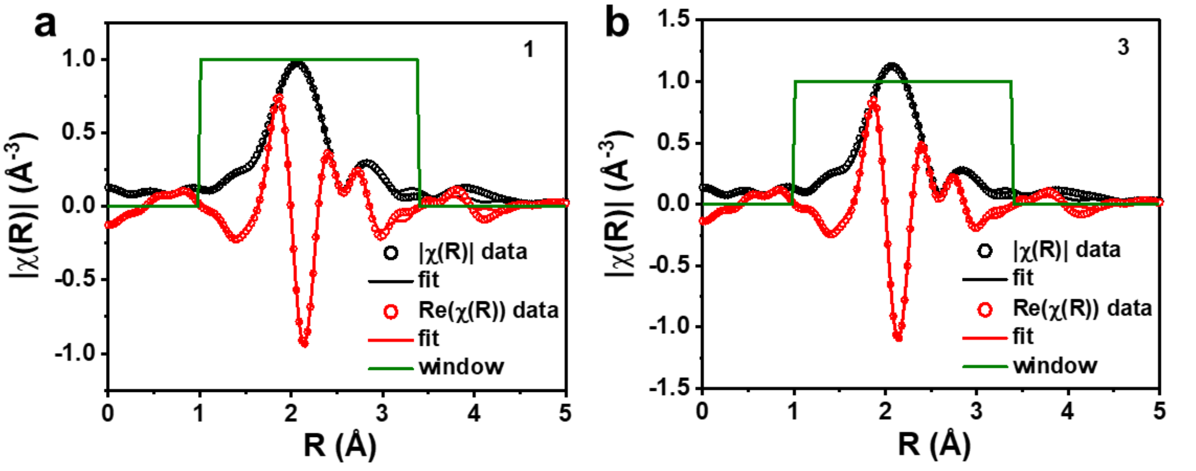

Fig. S13. Fitting to the R-space of the Ni K-edge EXAFS data for composites **1 and 3** showing the magnitude (black) and real components of R (red).

Table S5. Results of linear combination fitting to the Mo K-edge XANES.

Composite	MoO_2 Fraction	MoO_3 Fraction	Mo_2C Fraction
**1**	0.14 ± 0.02	0.34 ± 0.03	0.52 ± 0.05
**2**	0.17 ± 0.02	0.36 ± 0.03	0.47 ± 0.05
**3**	0.17 ± 0.02	0.29 ± 0.03	0.54 ± 0.05
**Ref.1**	0.11 ± 0.02	0.30 ± 0.03	0.59 ± 0.05
**Ref.2**	0.34 ± 0.03	0.66 ± 0.07	0.0 ± 0.01

Table S6. Results of the EXAFS fitting for the Mo–K and Ni-K edge data. Due to the low coordination and high correlation with the refinement of the coordination number, the σ^2 parameters for the Mo-C/O and Ni-Ni paths were fixed as refined from the MoO_3 and Ni metal reference materials.

Composite	Scattering Path	R (Å)	σ^2 (Å^2)	CN
**Mo-K**				
**1**	Mo-C/O	2.04 ± 0.02	0.002*	1.0 ± 0.2
	Mo-Mo	(2.93 – 3.00) ± 0.02	0.007 ± 0.002	3.8 ± 0.4
**2**	Mo-C/O	2.05 ± 0.02	0.002*	1.0 ± 0.2
	Mo-Mo	(2.93 – 3.00) ± 0.02	0.005 ± 0.002	5.0 ± 0.5
**3**	Mo-C/O	2.06 ± 0.02	0.002*	1.2 ± 0.2
	Mo-Mo	(2.93 – 3.00) ± 0.02	0.005 ± 0.001	6.0 ± 0.6
**Ni-K**				
**1**	Ni-O	2.04 ± 0.02	0.006 ± 0.002	3.4 ± 0.4
	Ni-Ni	2.45 ± 0.10	0.006*	0.6 ± 0.2
**2**	Ni-O	2.04 ± 0.02	0.006 ± 0.002	4.0 ± 0.4
	Ni-Ni	2.46 ± 0.10	0.006*	0.6 ± 0.3
**3**	Ni-O	2.04 ± 0.02	0.005 ± 0.002	3.5 ± 0.4
	Ni-Ni	2.47 ± 0.10	0.006*	0.7 ± 0.3

Table S7. Comparison of the OER activities of the recently reported highly active molybdenum carbide-based catalysts in alkaline conditions.

Catalysts	electrolyte	η_{10}/ mV	Tafel slope / mV dec^{-1}	Ref.
**Composite 2**	1 M KOH	320	68.8	This work
γ-MoC/Ni@NC	1 M KOH	310	62.7	[7]
Ni/Mo_2C-PC	1 M KOH	368	-	[8]
Ni/Mo_2C-NCNFs	1 M KOH	288	78.4	[9]
Co/β-Mo_2C@N-CNT	1 M KOH	356	67	[10]
Ni−MoxC/NC-100	1 M KOH	328	74	[11]
$Co_{0.1}$-β-Mo_2C@NC	1 M KOH	262.2	28.8	[12]
Mo_xCo_xC@C	1 M KOH	295	35	[13]
Mo_2C-NC@CoFe	1 M KOH	320	48	[14]
Co-Mo_2C	0.1 M KOH	347	38	[15]
Mo_2C/CS	1 M KOH	320	98	[16]
$(MoS_2)_{0.125}Mo_2C$	0.1 M KOH	280	209	[17]
B,N:Mo_2C@BCN	1 M KOH	290	61	[18]
Co SAs/Mo_2C	1 M KOH	270	74.9	[19]

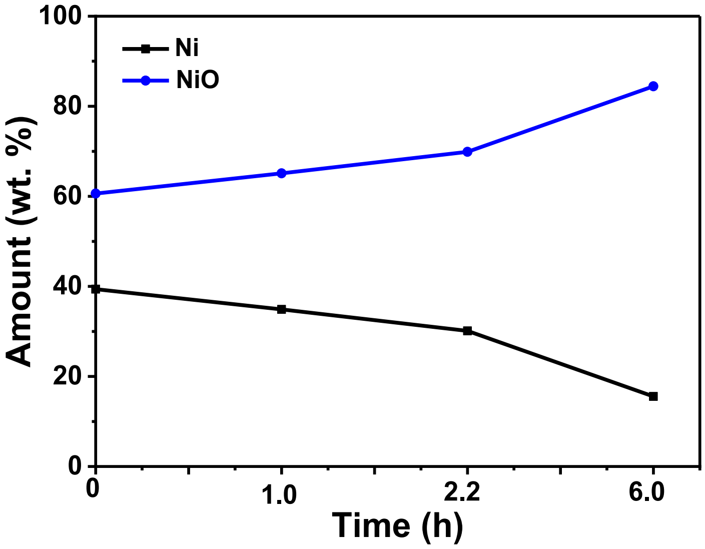

Fig. S14. Linear combination fitting results of Ni K-edge XANES using Ni and NiO after different time scale of CA measurements.

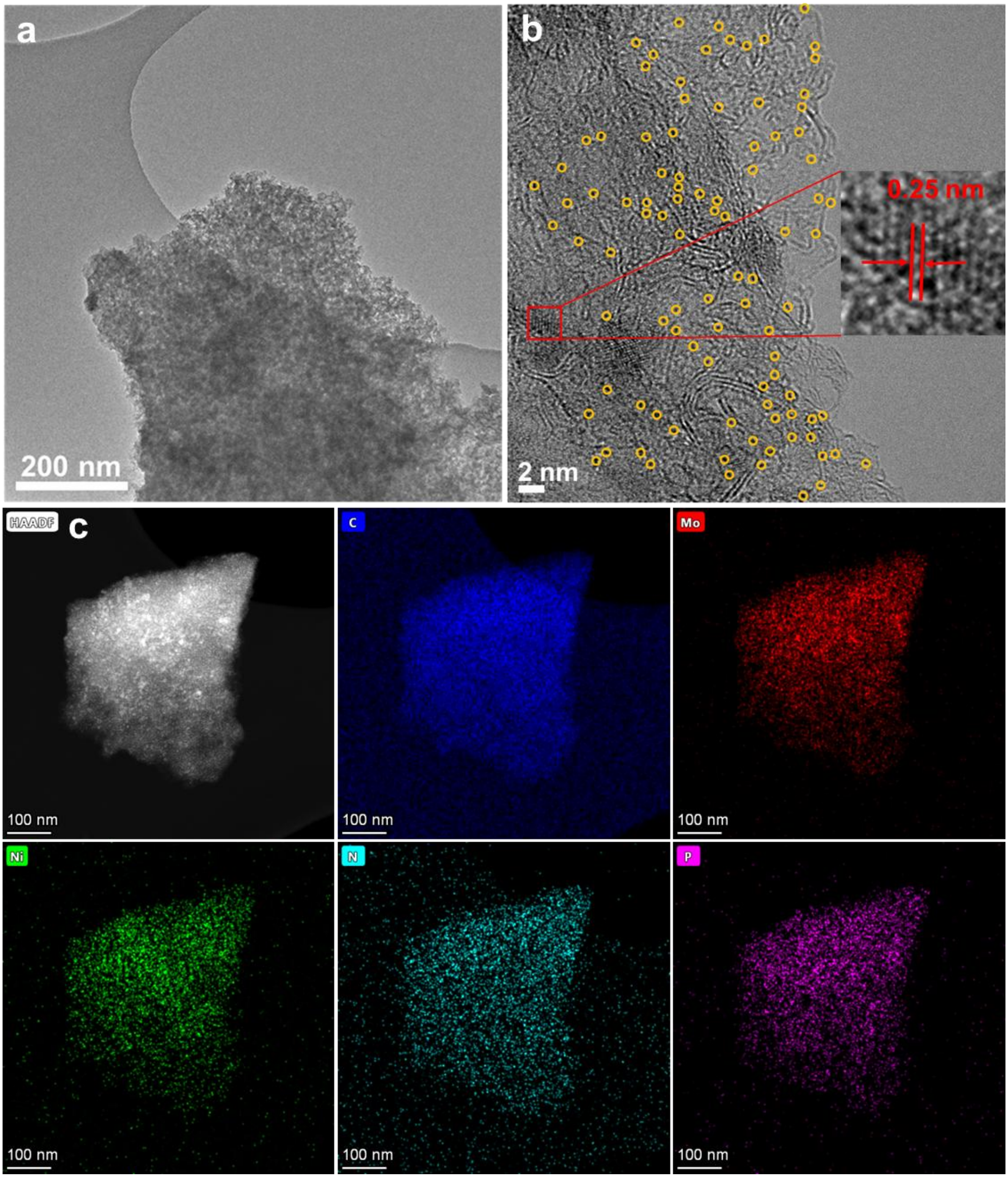

Fig. S15. (a) Overview TEM image and (b) AC-HRTEM image of **2** after CA test, the insert shows a magnified image of a nanocrystal. (c) HAADF-STEM and corresponding EDS mapping of **2**, showing the homogeneous dispersion of Mo, Ni, N and P on the mesoporous carbon. The lattice spacing of 0.25 nm corresponds to the (102) plane of η-MoC and/or (041) plane of MoO_3.

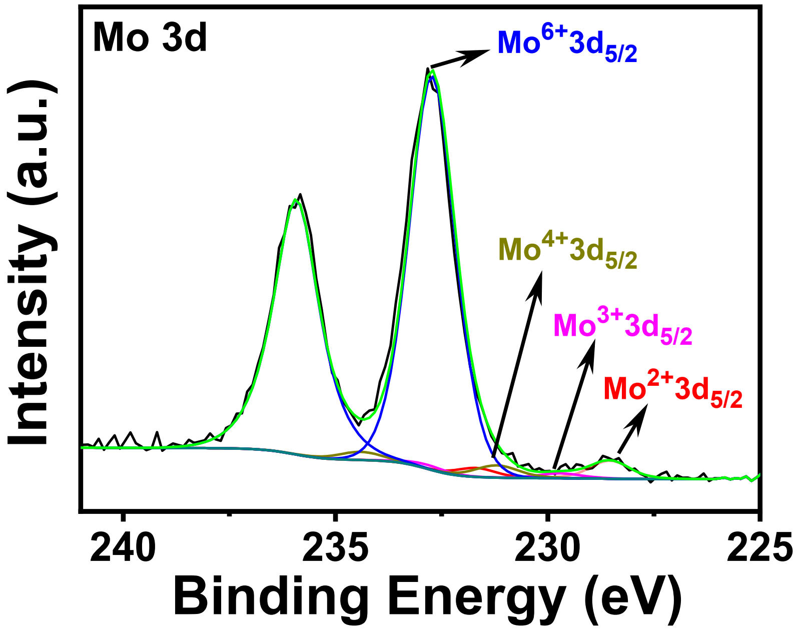

Fig. S16. High-resolution deconvoluted XPS spectra of Mo 3d for **2** after CA test.

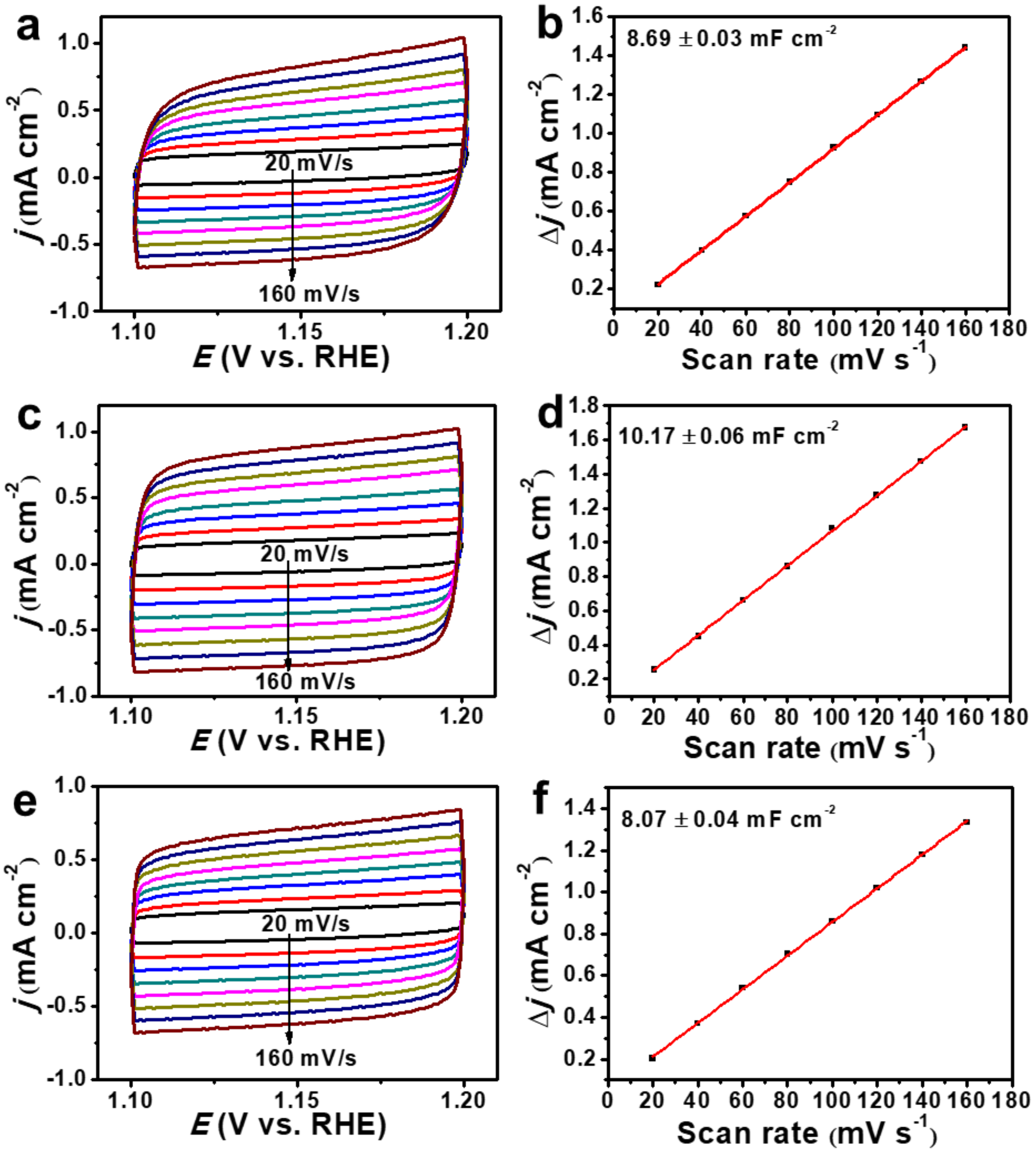

Fig. S17. ECSA study of the catalysts. (a,c,e) CV curves for **1**, **2** and **3** at various scan rates. (b,d,f) The corresponding linear fitting plots of differences in current density ($\Delta j = j_a - j_c$) at a potential of 1.15 V for **1**, **2** and **3** depending on the scan rate. j_a: anodic current density; j_c: cathodic current density.

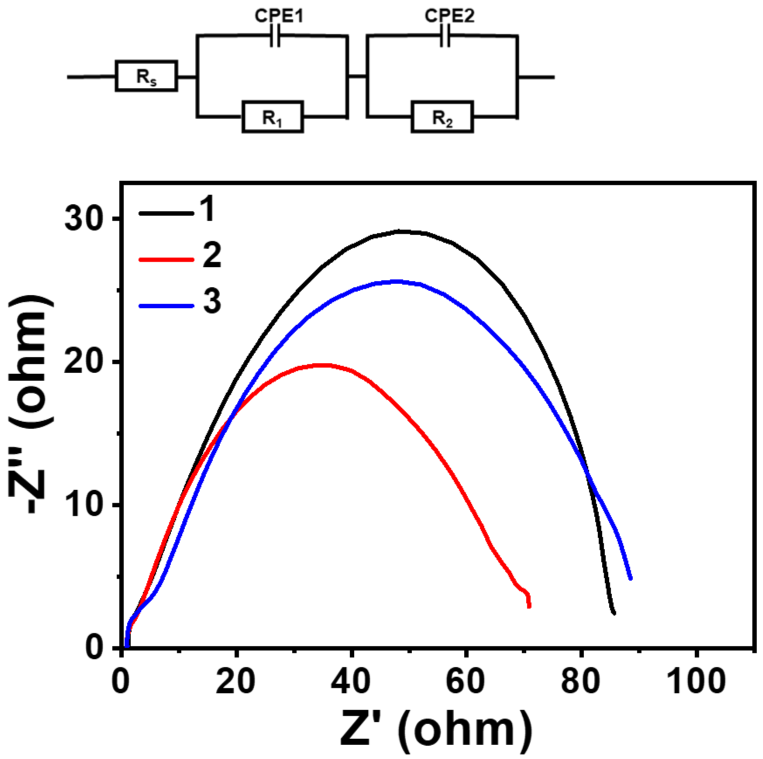

Fig. S18. Nyquist plots with frequency ranges from 1000 kHz to 0.01 Hz of **1, 2** and **3**. The equivalent circuit above the figure was used for fitting.

5. Supplementary References

1 A. Tahar, S. Benadji, T. Mazari, L. Dermeche, C. Marchal-Roch and C. Rabia, *Catal. Letters*, 2015, **145**, 569–575.

2 O. Müller, M. Nachtegaal, J. Just, D. Lützenkirchen-Hecht and R. Frahm, *J. Synchrotron Radiat.*, 2016, **23**, 260–266.

3 A. H. Clark, J. Imbao, R. Frahm and M. Nachtegaal, *J. Synchrotron Rad*, 2020, **27**, 551–557.

4 C. Tang, H. Zhang, K. Xu, Q. Zhang, J. Liu, C. He, L. Fan and T. Asefa, *J. Mater. Chem. A*, 2019, **7**, 18030–18038.

5 Z. Zhou, Z. Yuan, S. Li, H. Li, J. Chen, Y. Wang, Q. Huang, C. Wang, H. E. Karahan, G. Henkelman, X. Liao, L. Wei and Y. Chen, *Small*, 2019, **15**, 1–11.

6 Y. Huang, Q. Gong, X. Song, K. Feng, K. Nie, F. Zhao, Y. Wang, M. Zeng, J. Zhong and Y. Li, *ACS Nano*, 2016, **10**, 11337–11343.

7 X. Zhang, L. Huang, Y. Han, M. Xu and S. Dong, *Nanoscale*, 2017, **9**, 5583–5588.

8 Z. Y. Yu, Y. Duan, M. R. Gao, C. C. Lang, Y. R. Zheng and S. H. Yu, *Chem. Sci.*, 2017, **8**, 968–973.

9 M. Li, Y. Zhu, H. Wang, C. Wang, N. Pinna and X. Lu, *Adv. Energy Mater.*, 2019, **9**, 1803185.

10 T. Ouyang, Y. Q. Ye, C. Y. Wu, K. Xiao and Z. Q. Liu, *Angew. Chem. Int. Ed.*, 2019, **58**, 4923–4928.

11 D. Das, S. Santra and K. K. Nanda, *ACS Appl. Mater. Interfaces*, 2018, **10**, 35025–35038.

12 X. Zhu, X. Zhang, L. Huang, Y. Liu, H. Zhang and S. Dong, *Chem. Commun.*, 2019, **55**, 9995–9998.

13 C. Chen, A. Wu, H. Yan, Y. Xiao, C. Tian and H. Fu, *Chem. Sci.*, 2018, **9**, 4746–4755.

14 S. Wang, G. Bendt, S. Saddeler and S. Schulz, *Energy Technol.*, 2019, **7**, 1–11.

15 M. J. Kim, S. Kim, D. H. Song, S. K. Oh, K. J. Chang and E. A. Cho, *Appl. Catal. B: Environ*, 2018, **227**, 340–348.

16 H. Wang, Y. Cao, C. Sun, G. Zou, J. Huang, X. Kuai, J. Zhao and L. Gao, *ChemSusChem*, 2017, **10**, 3540–3546.

17 A. P. Tiwari, Y. Yoon, T. G. Novak, A. Azam, M. Lee, S. S. Lee, G. hyoung Lee, D. J. Srolovitz, K. S. An and S. Jeon, *Adv. Mater. Interfaces*, 2019, **6**, 1900948.

18 M. A. R. Anjum, M. H. Lee and J. S. Lee, *ACS Catal.*, 2018, **8**, 8296–8305.

19 Z. Kou, W. Zang, W. Pei, L. Zheng, S. Zhou, S. Zhang, L. Zhang and J. Wang, *J. Mater. Chem. A*, 2020, **8**, 3071–3082.
